# Unveiling the vital role of OGG1 in inflammation, vascular endothelial damage, and cell death in obstetric and gynecological diseases

**DOI:** 10.1007/s13577-025-01268-x

**Published:** 2025-08-14

**Authors:** Yang Li, Wenying Zhang, Bo Wang, Fuju Wu

**Affiliations:** Department of Gynecology and Obstetrics, The Second Hospital of Jilin University, Changchun, 130041 People’s Republic of China

**Keywords:** OGG1, Inflammation, Vascular endothelial damage, Cell death, Obstetric and gynecological diseases

## Abstract

The DNA repair enzyme 8-oxoguanine DNA glycosylase-1 (OGG1) plays a crucial role in the initiation of DNA base excision repair pathway by recognizing and excising the oxidative base lesions including 7,8-dihydro-8-oxoguanine (8-oxoG). Beyond its canonical function in DNA repair, OGG1 has been implicated in regulating inflammation-related genes, growth factor expression, and various cell death pathways, including apoptosis, parthanatos, and autophagy. These mechanisms are often involved in obstetric and gynecological disorders, which are frequently characterized by inflammation, endothelial dysfunction, and dysregulated cell death. As such, OGG1 emerges as a potential therapeutic target for these conditions. However, comprehensive reviews detailing OGG1’s mechanistic roles in reproductive diseases remain scarce. This review aims to synthesize current knowledge primarily on non-canonical functions of OGG1, with a focus on its potential involvement in disorders such as endometriosis, polycystic ovary syndrome, uterine fibroids, and malignancies, and to highlight its promise as a therapeutic target.

## Introduction

Reactive free radicals, including superoxide (O•^−2^), hydrogen peroxide (H_2_O_2_), hydroxyl radicals (•OH), and peroxynitrite (ONOO^−^), can damage proteins, lipids, DNA, and RNA within cells, ultimately leading to dysfunction and cell death [1, 2]. When DNA is oxidized, it can undergo various types of damage, including base modifications (oxidized bases), strand breaks (both single and double strand), and cross-linking between strands [3]. Among all nucleic acid bases, guanine has the lowest oxidation potential, making it particularly vulnerable to reactive oxygen species (ROS). This susceptibility leads to the formation of 7,8-dihydro-8-oxoguanine (8-oxoG), a well-established marker of oxidative stress. The accumulation of 8-oxoG in DNA is considered one of the most reliable indicators of oxidative damage [4] and is frequently detected in guanine-rich promoter regions of genes [5]. Oxidative base modifications induced by ROS contribute to a variety of biological and pathological processes, including mutagenesis, carcinogenesis, neurodegeneration, and aging, thereby underscoring the central role of oxidative stress in disease development and cellular dysfunction [6–9].

The DNA repair enzyme 8-oxoguanine DNA glycosylase-1 (OGG1) primarily recognizes and excises the prominent oxidative lesion 8-oxoG in double-stranded DNA via the base excision repair (BER) pathway [10]. Additionally, OGG1 also exhibits significant activity against other oxidatively damaged bases, including 2,6-diamino-4-hydroxy-5-formamidopyrimidine (FapyG) and 8-oxo-7,8-dihydroadenine (8-oxoA), though with reduced efficiency for 8-oxoA compared to 8-oxoG [11–13]. The BER pathway serves as the primary mechanism responsible for repairing small, non-bulky oxidative DNA lesions. OGG1 and MUTYH (MutY DNA glycosylase) are core enzymes in the 8-oxoG repair pathway. OGG1 excises 8-oxoG lesions, while MUTYH removes adenines misincorporated opposite unrepaired 8-oxoG during replication. Their collaboration prevents G:C → T:A transversions induced by oxidative damage [14, 15]. By excising mutagenic 8-oxoG lesions from mtDNA, mitochondrial OGG1 protein safeguards genome integrity. This repair prevents mtDNA damage-driven ROS overproduction, thereby indirectly supporting cellular redox homeostasis [16]. Besides mitochondria, oxidoreductases such as NADPH oxidases and cytochrome P450 enzymes also generate ROS, contributing to redox signaling and cellular homeostasis. These ROS act as second messengers in gene expression, immunity, and aging, while their dysregulation is linked to cancer, cardiovascular disease, and neurodegeneration [17–19].

RNA molecules exhibit heightened susceptibility to ROS-induced oxidative damage due to converging factors: their single-stranded topology (exposing nucleobases), absence of histone shielding, rapid metabolic turnover, and proximity to endogenous ROS sources (e.g., mitochondrial electron transport chain). Moreover, the levels of 8-oxoG in RNA are 14–25 times higher than those in double-stranded DNA [20]. When mRNA is heavily oxidized, cells degrade the damaged transcripts via RNA degradation, while extensive damage at the tissue level may lead to apoptosis, respectively reflecting localized and systemic responses to oxidative stress [8, 21]. When mRNA is heavily oxidized, cells typically degrade the damaged transcripts through RNA degradation mechanisms to preserve transcriptomic integrity. However, under sustained or severe oxidative stress at the tissue level, extensive cell injury or death may occur, leading to apoptosis and the subsequent release of oxidized nucleic acids—such as 8-oxoG-modified RNA and DNA—into the bloodstream. These extracellular oxidized nucleic acids have been proposed as potential biomarkers for oxidative stress-related conditions, including autoimmune disorders, diabetes, and cardiovascular diseases [22].

Oxidative stress is a key factor in the development and progression of several gynecological and obstetric conditions. Specifically, it contributes to diseases such as polycystic ovary syndrome [23], endometriosis [24], preeclampsia [25], preterm birth [26], ovarian cancer [27], and cervical cancer [28] through mechanisms including cellular damage, inflammatory responses, and apoptosis. Elevated ROS levels can damage DNA, leading to genetic mutations and dysfunction, which further exacerbate the pathological processes underlying these diseases. Obstetric and gynecological diseases typically involve complex pathophysiological processes, including inflammation, vascular endothelial damage, and cell death. Although OGG1 plays a crucial role in various physiological and pathological processes, comprehensive reviews summarizing its mechanistic role in obstetric and gynecological diseases remain limited. This lack of a thorough exploration underscores the need for further research to better understand the therapeutic potential of OGG1 for these conditions. In this review, the mechanisms by which OGG1 regulates inflammation, vascular endothelial damage, and cell death are summarized, emphasizing its involvement and potential as a therapeutic target in obstetric and gynecological diseases.

## Role of OGG1 in cellular pathophysiological processes

OGG1 plays a critical role in several biological processes that contribute to the pathophysiology of obstetric and gynecological diseases, particularly through the regulation of immune and inflammatory responses, vascular endothelial damage, and cell death. Through its interaction with oxidative DNA lesions, particularly 8-oxoG modulates immune and inflammatory signaling by activating pathways such as NF-κB and mitogen-activated protein kinases (MAPK). OGG1 also influences vascular endothelial function by influencing the expression of genes such as vascular endothelial growth factor (VEGF). In addition, OGG1 plays a critical role in multiple cell death pathways—including apoptosis, parthanatos, and autophagy—thereby underscoring its broad influence on cellular fate and survival. Collectively, these findings underscore the significance of OGG1 as a potential therapeutic target in diseases characterized by oxidative stress and chronic inflammation.

### Immune inflammation hyperactivation

**Fig. 1:**
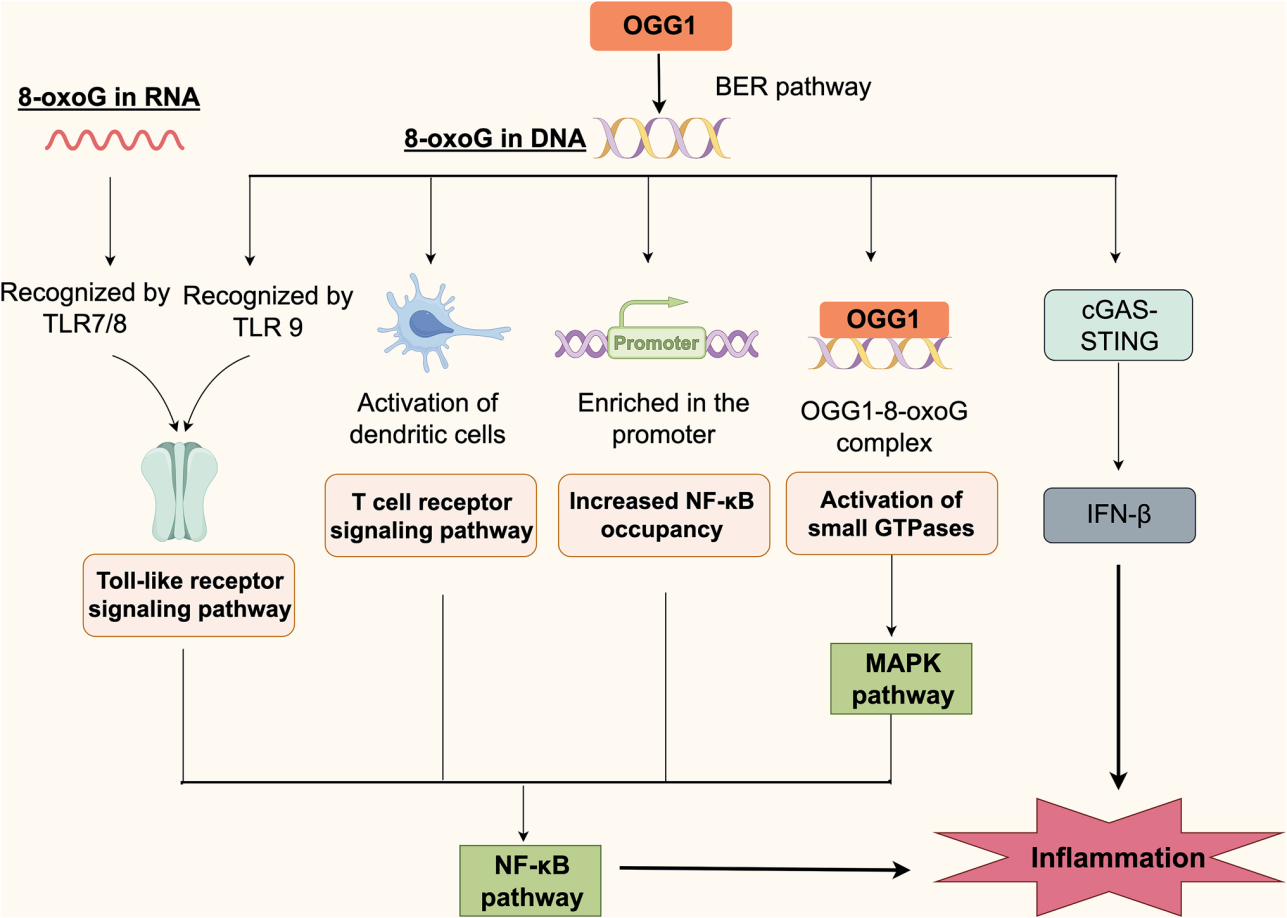
OGG1-mediated inflammatory signaling pathways triggered by 8-oxoG. OGG1 recognizes and excises 8-oxoguanine (8-oxoG) via the base excision repair (BER) pathway. Depending on the form and location of 8-oxoG, distinct immune signaling cascades are initiated. 8-oxoG in RNA is recognized by TLR7/8, while 8-oxoG in DNA is detected by TLR9, activating Toll-like receptor signaling and NF-κB pathways. In dendritic cells, 8-oxoG enhances T cell receptor signaling and pro-inflammatory cytokine release. OGG1 binding to promoter-enriched 8-oxoG facilitates NF-κB recruitment and transcriptional activation of inflammatory genes. The OGG1–8-oxoG complex also activates small GTPases (e.g., Ras), triggering the MAPK pathway. In addition, OGG1 modulates the cGAS–STING axis to regulate IFN-β expression. Together, these pathways converge to amplify inflammation under oxidative stress

When tissues or cells are exposed to pathogens or environmental factors, the rapid elevation of ROS levels triggers oxidative stress, serving as a critical innate immune defense mechanism. The expression of pro-inflammatory cytokines and chemokines is highly dependent on the activation of ROS signaling pathways [29]. 8-oxoG significantly influences the immune system by upregulating the expression of cell surface molecules and promoting the secretion of pro-inflammatory mediators in human monocyte-derived dendritic cells (moDCs), a process that is dependent on OGG1 [30, 31]. 8-oxoG can also be recognized by Toll-like receptors (TLRs) [31–34]; however, blockade of TLR-mediated signaling with the MyD88-specific inhibitory peptide does not affect 8-oxodG-induced activation of moDCs, suggesting that excluding the involvement of endosomal TLRs in this process [30]. Under oxidative stress, 8-oxoG accumulates in the promoter regions of pro-inflammatory genes, facilitating NF-κB binding through OGG1 and thereby inducing the expression of inflammatory mediators [35–37]. Additionally, the OGG1-8-oxoG complex activates Ras proteins, which subsequently stimulate the MAPK signaling pathway, leading to increased expression of inflammatory cytokines and the initiation of inflammatory responses [38]. Small GTPases, including Rac1 and RhoG, also contribute to this process, further modulating dendritic cell functions [39]. Visnes et al. demonstrated that the small-molecule inhibitor TH5487 targets OGG1, resulting in decreased expression of pro-inflammatory genes and attenuation of inflammation, including reduced immune cell recruitment in the lungs of mice [40]. These findings suggest that inhibition of OGG1’s substrate binding may mitigate inflammatory responses in vivo (Fig. 1).

In study[30], the authors used two independent models to investigate the immunomodulatory effects of 8-oxoG: a murine model of allergic airway inflammation and in vitro experiments with human moDCs. Intranasal 8-oxoG administration in mice elevated dendritic cell-related gene expression and serum ovalbumin-specific IgE levels. Similarly, 8-oxoG exposure in human moDCs upregulated surface markers (CD40, CD86, CD83, HLA-DQ) and pro-inflammatory cytokines (IL-6, TNF, IL-8), with minimal effect on IL-10. These responses were abolished by OGG1 knockdown, indicating that 8-oxoG, generated via OGG1-mediated base excision repair, promotes dendritic cell activation under oxidative stress. Additionally, extracellular mitochondrial DNA containing high levels of 8-oxoG serves as a potent immunostimulant for plasmacytoid dendritic cells [31]. Collectively, these findings indicate that 8-oxoG released from ROS-induced DNA damage plays a critical role in immune activation.

TLRs are innate immune receptors expressed on leukocytes, epithelial cells, and notably on placental immune cells and chorionic trophoblasts. TLR7, TLR7/8, and TLR9 recognize various guanosine derivatives associated with the 8-oxoG structure, as well as 8-oxoG-containing oligodeoxynucleotides, both of which activate immune cells [31–34]. Endosomal TLRs utilize the MyD88 adaptor protein to initiate downstream signaling cascades that activate the NF-κB pro-inflammatory pathway [41]. However, blockade of TLR-mediated signaling with the MyD88-specific inhibitory peptide did not affect 8-oxodG-induced activation of moDCs, thereby excluding the involvement of endosomal TLRs in 8-oxoG-triggered moDC activation [30].

In mouse embryonic fibroblasts exposed to oxidative conditions, biotin-labeled 8-oxoG-containing genomic fragments were isolated and sequenced, revealing that 8-oxoG is enriched in promoter regions, 5′ untranslated regions (5′ UTRs), and 3′ UTRs [5]. Notably, the peak expression of pro-inflammatory genes coincides with both the highest intracellular ROS levels and maximal accumulation of 8-oxoG in genomic regulatory regions, suggesting that OGG1 may promote transcriptional activation through a non-catalytic mechanism [35, 42, 43]. High levels of 8-oxoG recruit substantial amounts of OGG1 to gene promoters. Although the glycosylase activity of OGG1 is inhibited under stress conditions, its binding induces DNA distortion that facilitates the recruitment of NF-κB/RelA to cis-acting elements. This, in turn, promotes the assembly of transcriptional machinery, including transcription factors such as SP1, transcription initiation factor II-D, and RNA polymerase II, ultimately initiating gene transcription [35–37]. In this process, 8-oxoG is not only a base-damage product but also a ligand for OGG1, co-recruiting transcription factors and facilitating the assembly of the transcription machinery.

OGG1 binds its repair target, 8-oxoG, outside its catalytic center, forming an OGG1–8-oxoG complex. This complex may function as a guanosine exchange factor, interacting with small GTPases, such as Ras (K-Ras, N-Ras, and H-Ras) and promoting the exchange of GDP for GTP. Activation of Ras proteins subsequently leads to the phosphorylation of MAPKs (MEK1/2 and ERK1/2), thereby initiating the MAPK signaling pathway [38]. Further, OGG1-mediated BER not only maintains genomic stability but also initiates pro-inflammatory signaling in airway epithelial cells through 8-oxoG-dependent activation of the KRAS/MAPK/PI3K/NF-κB pathway, highlighting a novel link between DNA repair and innate immune responses [39]. Additionally, small GTPases can either positively or negatively regulate NF-κB activation, depending on the cellular context [44]. Emerging evidence indicates that small GTPases, such as Rac1 and RhoG, modulate gene expression and activate the NF-κB signaling pathway via the MAPK and PI3K cascades [45–47]. Small GTPases also contribute to the regulation of various dendritic cell functions, including differentiation [48, 49], endocytosis [50], maturation [51, 52], chemotaxis [50], antigen presentation [50], cross-presentation [53–55], and T cell activation [56].

OGG1 also promotes inflammation through additional signaling pathways. In OGG1-knockout (*Ogg1*^*−/−*^) mice, the expression of the type I interferon (IFN) gene *Ifnβ* and downstream interferon-stimulated genes (ISGs), including *Isg15* and *Irf9*, is significantly increased [57]. Moreover, OGG1 regulates *Ifnβ* expression through the cGAS–STING signaling pathway [57]. Additionally, signal transducer and activator of transcription 1 (STAT1) is essential for mediating endotoxin-induced OGG1 expression and the resulting inflammatory response. Upon endotoxin exposure, OGG1 functions as a coactivator of STAT1, enhancing its transcriptional activity and promoting the expression of pro-inflammatory mediators at the gene level [58].

### Vascular endothelial damage

**Fig. 2:**
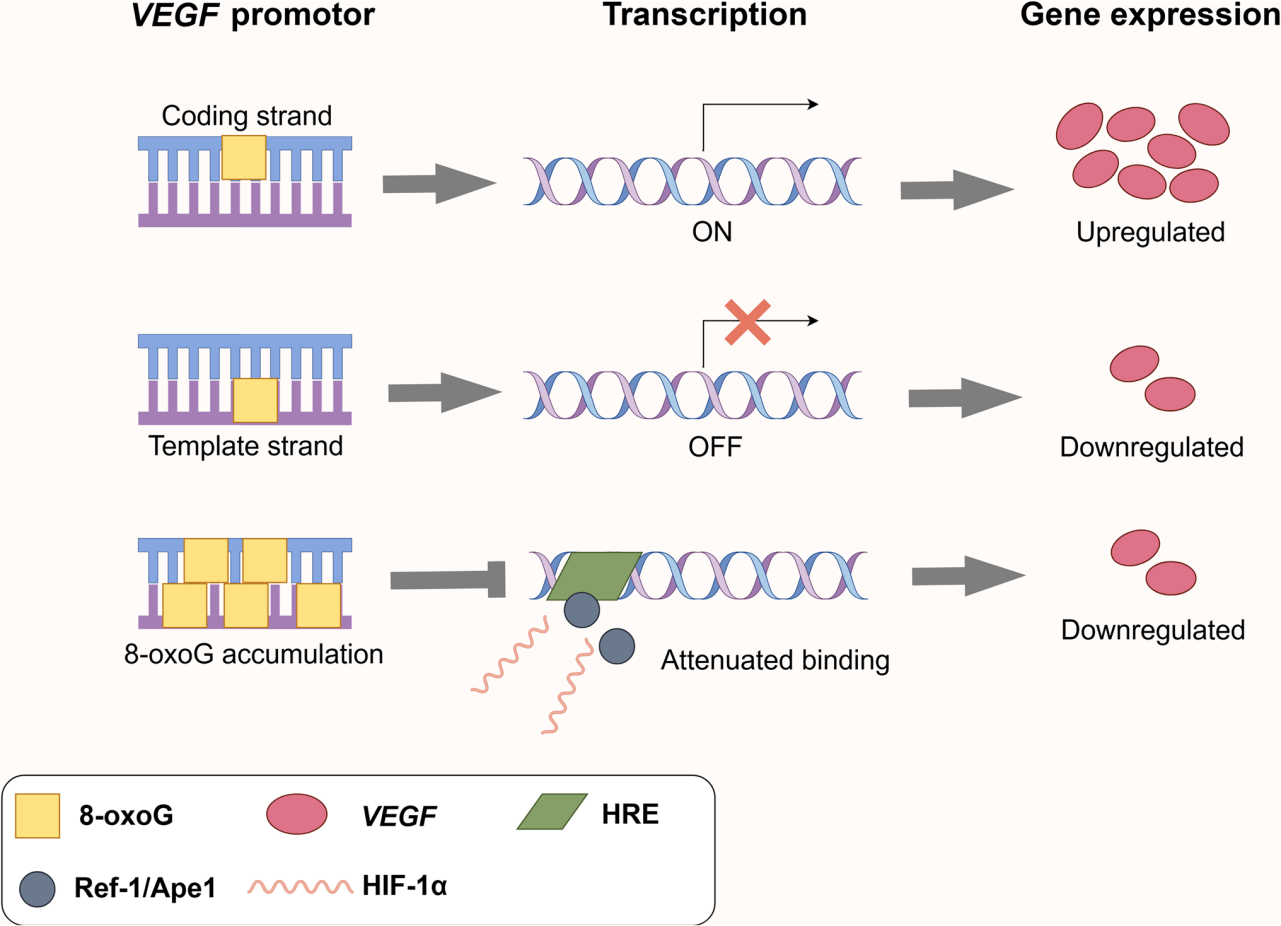
OGG1-mediated 7,8-dihydro-8-oxoguanine (8-oxoG) repair and *VEGF* transcriptional modulation. When OGG1 targets the coding strand of the *VEGF* promoter, transcription is activated, resulting in the upregulation of *VEGF* expression. Conversely, when OGG1 binds to the template strand, transcription is inhibited, leading to the downregulation of gene expression. The accumulation of 8-oxoG in the *VEGF* promoter region impairs the binding of transcription factors, such as Ref-1/Ape1 and HIF-1α, reducing their binding efficiency. This inhibition of transcription results in decreased *VEGF* expression

On human chromosomes, G-quadruplex-forming sequences (G4-forming sequences) are predominantly enriched in the promoter regions and 5′ UTRs of highly expressed genes, particularly those associated with cancer or amplified in somatic cells, such as *MYC* [59, 60]. The promoter region of the *VEGF* gene is guanine rich and contains potential G-quadruplex-forming sequences (PQSs), which can fold into G-quadruplex structures and regulate gene transcription [61–63]. ROS preferentially target guanine-rich *VEGF* promoter region, oxidizing guanine to 8-oxoG. The resulting 8-oxoG lesions are recognized and excised by OGG1, leading to the formation of apurinic/apyrimidinic (AP) sites. These AP sites may cause double-strand DNA breaks, exposing PQSs to unfolding into G-motifs and altering chromatin structure. This structural change facilitates the recruitment of transcriptional activators, such as hypoxia-inducible factor 1-α (HIF1-α), ultimately enhancing the transcription of *VEGF* and related genes [64, 65]. Pastukh et al. demonstrated that the formation of 8-oxoG within hypoxia response elements (HREs) coincides with the recruitment of HIF-1α, OGG1, and redox effector factor-1 (Ref-1)/apurinic/apyrimidinic endonuclease 1 (Ape1), as well as the induction of DNA strand breaks. Silencing of OGG1 impairs BER, increases 8-oxoG accumulation, and reduces HIF-1α and Ref-1/Ape1 binding, ultimately leading to diminished *VEGF* expression [66]. 8-oxoG is not only a marker of oxidative DNA damage but also exerts strand-specific regulatory effects on gene transcription. Dr. Fleming et al. site-specifically incorporated 8-oxoG into the PQS region of the human *VEGF* promoter and found that its position on the coding (sense) or template (antisense) strand determines its impact on transcription [67]. When located on the coding strand, 8-oxoG promotes gene expression via the OGG1-mediated BER pathway. In contrast, when situated on the template strand, it represses transcriptional activity through a BER-independent mechanism [67]. Together, these findings suggest that under oxidative stress, the formation of G-quadruplexes and 8-oxoG, along with OGG1-mediated repair, regulates *VEGF* gene expression by altering chromatin structure and transcription factor recruitment at its promoter region (Fig. 2).

### Cell death

**Fig. 3:**
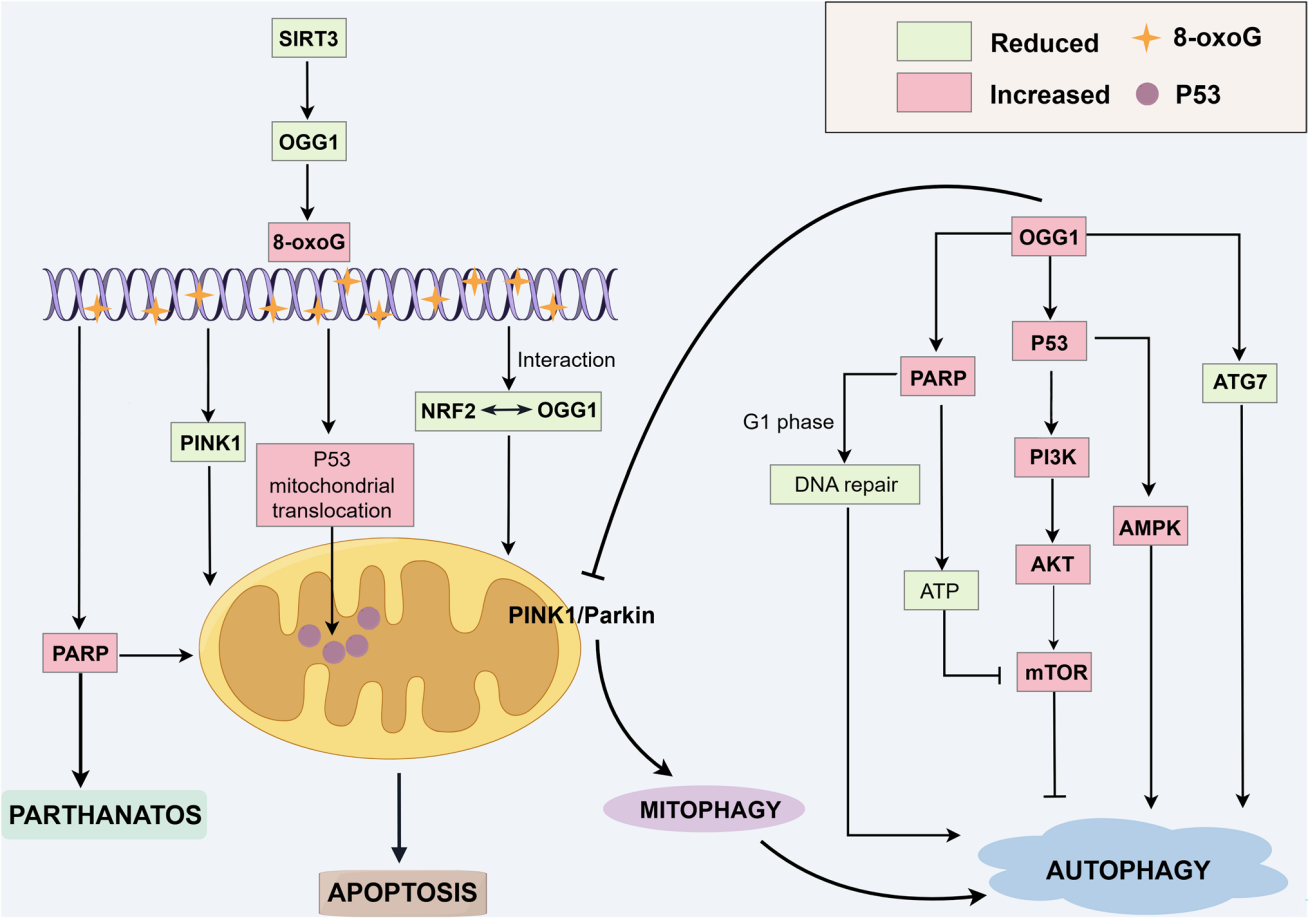
Regulatory role of OGG1 in cell death pathways. The reduction of Sirt3 leads to decreased OGG1 levels, resulting in the accumulation of 7,8-dihydro-8-oxoguanine (8-oxoG), activation of the PARP pathway, inhibition of PINK1, and enhanced mitochondrial translocation of p53. These events collectively disrupt mitochondrial homeostasis and trigger apoptosis. Furthermore, OGG1 accumulation is linked to PARP1 activation, which induces parthanatos, a form of PARP-dependent cell death. In addition to its role in apoptosis, OGG1 promotes autophagy by cooperating with p53 to activate the PI3K/AKT and AMPK pathways while inhibiting mTOR. OGG1 also facilitates autophagy by binding to proteins that mediate G1-phase DNA damage repair mechanisms, reducing ATP production, inhibiting the PINK1/Parkin pathway, and downregulating Atg7 expression

The role of OGG1 in cell death is primarily mediated through its DNA repair function, modulation of oxidative stress and inflammation, and maintenance of mitochondrial integrity. As such, alterations in OGG1 expression or function can significantly affect cell survival and death under various pathological conditions (Fig. 3).

#### Apoptosis

Apoptosis is a programmed cell death process initiated by various intracellular and extracellular signals that ultimately lead to systematic cellular demise. If oxidative DNA damage is not efficiently repaired, it can accumulate and activate apoptotic pathways. OGG1 is essential for maintaining mitochondrial homeostasis by repairing oxidatively damaged mitochondrial DNA, regulation of mitochondrial function, and prevention of apoptosis. Mitochondria-targeted human α-hOgg1 (mt-hOgg1) protects cells against oxidative stress-induced mitochondrial dysfunction and apoptosis by acting as a chaperone protein for mitochondrial aconitase, independent of its DNA repair activity [68]. Sheng et al. discovered that the buildup of 8-oxoG in neuronal mitochondrial DNA leads to neuronal loss through a mechanism dependent on calpain activity. Additionally, the delayed accumulation of 8-oxoG in microglial nuclei led to PARP-dependent activation of apoptosis-inducing factor (AIF), which in turn exacerbated microgliosis [69]. Cheng et al. also demonstrated that Sirtuin 3 (Sirt3)-mediated regulation of OGG1 acetylation and turnover is critical for mitochondrial DNA repair, mitochondrial integrity, and resistance to oxidative stress-induced apoptosis. Deletion of Sirt3 increases OGG1 acetylation, which not only enhances its repair activity but also promotes its degradation, ultimately impairing mitochondrial function and exacerbating apoptosis under oxidative stress [70]. Kim et al. found that maintenance of mtOGG1 in alveolar epithelial cells is crucial for preventing PTEN-induced putative kinase 1 (PINK1) deficiency, which contributes to apoptosis and lung fibrosis [16]. In a previous study, the same group demonstrated that mt-hOgg1 and aconitase-2 protect alveolar epithelial cells against oxidant-induced apoptosis by preserving mitochondrial function, inhibiting p53 mitochondrial translocation, and suppressing intrinsic apoptotic signaling [71]. Additionally, Nrf2 binds to antioxidant response elements within the *Ogg1* promoter region, playing a critical role in mitochondrial DNA repair and the maintenance of mitochondrial homeostasis [72]. Collectively, these findings underscore the pivotal role of OGG1 in preserving mitochondrial integrity, regulating apoptosis, and protecting cells from oxidative stress under various physiological and pathological conditions.

#### Parthanatos

In 2007, Ted Dawson and Valina Dawson at the Johns Hopkins University School of Medicine identified a novel form of programmed cell death in neurons. They named this process parthanatos, which is a form of cell death dependent on poly(ADP-ribose) polymerase 1 (PARP1). Parthanatos is triggered by DNA damage followed by PARP1 activation. This newly identified form of cell death has gained significant attention in recent years and is now recognized as a key contributor to the pathogenesis of various degenerative diseases, including diabetes mellitus, Parkinson’s disease, and stroke [73–76]. Parthanatos is initiated by severe DNA damage, which leads to PARP1 hyperactivation [77, 78]. Ba et al. found that OGG1-initiated BER prevents the accumulation of mutagenic guanine base lesions and promotes cell survival under mild oxidative stress and low levels of 8-oxoG. However, under excessive oxidative stress, intermediates generated during the OGG1–BER process—including AP sites and strand breaks—serve as substrates for the activation of the DNA repair effector protein PARP1. Furthermore, excessive activation of PARP1 triggers AIF-mediated, caspase-independent cell death known as parthanatos [79]. Moreover, ROS inducers trigger parthanatos in cervical cancer cells through excessive excision of 8-oxoG mediated by OGG1, leading to extensive DNA strand breaks [80].

#### Autophagy

Whereas autophagy primarily functions as a survival mechanism, it can also contribute to cell death under specific conditions. OGG1 affects autophagy by interacting with specific proteins or genes that are part of the autophagy pathway. Apoptosis and autophagy, through shared and interconnected signaling pathways, may function independently or synergistically to determine cell survival or death in response to cellular stress and injury. p53 modulates both the expression and activity of OGG1, and its absence inhibits OGG1 from initiating BER [81]. The p53 protein is also crucial for DNA damage-induced apoptosis and can bidirectionally regulate autophagy depending on its subcellular localization [82]. It also induces autophagy by activating AMPK, suppressing mTOR activity, and enhancing the transcription of genes that regulate autophagy in response to damage [83, 84]. Hooten et al. found that OGG1 directly interacts with PARP1 through its N-terminal region and that this interaction is strengthened by oxidative stress [85]. PARP1 and OGG1 function within the same regulatory pathway, where the activity of PARP1 is essential for the repair of oxidative DNA damage mediated by OGG1 in G1 phase-arrested cells [86]. Additionally, PARP1 triggers ATP depletion and suppresses the mTOR pathway, thus controlling the initiation of autophagy. PARP1 is also vital for the successful induction of autophagy during starvation [87]. PINK1 is a serine/threonine protein kinase that localizes to mitochondria. Full-length PINK1 associates with the pro-autophagy protein Beclin 1, thereby promoting autophagy [88]. Zhao et al. demonstrated that OGG1 exacerbates renal ischemia–reperfusion injury by suppressing the PINK1/Parkin-mediated mitophagy pathway and that OGG1 knockout or inhibition can mitigate this injury by activating mitophagy [89]. Wu et al. also reported that inhibiting OGG1 alleviates pulmonary fibrosis by blocking M2 macrophage polarization and initiating mitophagy mediated by PINK1 [90]. Ye et al. further discovered that OGG1 deficiency suppresses autophagy in both in vitro and in vivo models under hyperoxic conditions, as evidenced by reduced LC3-I to LC3-II conversion, diminished LC3 puncta, and decreased Atg7 expression [91]. Furthermore, OGG1 binds to the promoter of *Atg7* and suppresses the expression of this gene. Therefore, OGG1 influences autophagy through its interactions with proteins such as p53, PARP1, and PINK1.

## Role of OGG1 in obstetric and gynecological diseases

OGG1 has been implicated in the pathophysiology of several obstetric and gynecological diseases, including polycystic ovary syndrome, endometriosis, preeclampsia, preterm birth, and gynecologic cancers. In these conditions, oxidative stress and inflammation are central pathological features, with OGG1 contributing to cellular processes such as DNA repair, apoptosis, and regulation of inflammatory responses. The involvement of OGG1 in these diseases suggests its potential as both a biomarker and a therapeutic target, although further research is required to fully elucidate its role in these pathologies.

### Polycystic ovary syndrome

Polycystic ovary syndrome is the most common reproductive endocrine disorder among women, characterized by a complex pathophysiology and a wide range of clinical manifestations. Oxidative stress and chronic low-grade inflammation are key pathogenic processes involved in polycystic ovary syndrome, significantly affecting ovarian function and contributing to metabolic abnormalities [92, 93]. Several studies have reported significantly elevated levels of pro-inflammatory cytokines (e.g., IFN-γ, IL-2, TNF-α, IL-6, and IL-23) within the follicular fluid of patients with polycystic ovary syndrome [94, 95]. Furthermore, Xia et al. reported significantly elevated OGG1 protein expression in granulosa cells from PCOS patients, and also measured higher OGG1-related signal levels in serum and follicular fluid using ELISA, although the precise extracellular form and functional relevance of OGG1 in these fluids remains to be clarified [96]. Increased apoptosis in granulosa cells is a key pathogenic feature of ovaries affected by pathological ovary syndrome. This increase can impair follicular development, reduce oocyte quality, and affect the success rates of in vitro fertilization–embryo transfer (IVF-ET) procedures [97]. The administration of TH5487, a functional inhibitor of OGG1, leads to increased apoptosis and ROS production in ovarian granulosa cells, accompanied by a reduction in their inflammatory responses. These findings suggest that OGG1 may contribute to the pathogenesis of polycystic ovary syndrome by promoting activation of the NF-κB pathway and increasing IL-6 secretion [96]. This evidence further supports the anti-apoptotic and pro-inflammatory roles of OGG1 in the pathogenesis of polycystic ovary syndrome. However, the precise role of OGG1 in Polycystic ovary syndrome remains to be fully elucidated.

### Endometriosis

Endometriosis is a reproductive disorder in women characterized by the ectopic growth of endometrial cells and tissues outside the uterus. It is associated with ectopic endometrial tissue, immune system alterations, imbalances in cell growth and death, abnormal hormone signaling, and genetic factors [98, 99]. Carvalho et al. reported significantly increased levels of lipid peroxides and protein carbonyls in the peritoneal fluid of patients with endometriosis. Additionally, immunohistochemical analysis revealed elevated 8-OHdG levels, whereas OGG1 expression was significantly reduced, indicating impaired oxidative DNA repair capacity [100]. These findings suggest that oxidative cell damage is a significant factor underlying the progression of endometriosis. NF-κB promotes the growth and persistence of endometriotic cells by regulating angiogenesis, invasion, proliferation, and inflammation, thereby contributing to the progression of endometriosis [101]. An imbalance between estrogen, which promotes endometrial growth, and progesterone, which counteracts its effects and promotes differentiation, leads to impaired uterine function, immune cell infiltration, and inflammation. Additionally, aberrant angiogenesis and impaired apoptosis are key pathophysiological factors driving the progression of this condition [102]. A study by Siracusa et al., using a rat model of endometriosis induced by uterine debris injection, demonstrated that rapamycin-triggered autophagy and mitophagy result in enhanced apoptosis and reduced angiogenesis, leading to smaller lesion volumes, areas, and diameters [103]. Given the involvement of NF-κB, dysregulated angiogenesis, apoptosis, and autophagy in the pathophysiology of endometriosis, it can be inferred that OGG1 may promote inflammation and autophagy while inhibiting angiogenesis. However, this hypothesis requires further experimental validation.

### Preeclampsia

Preeclampsia is a progressive multisystem disorder of pregnancy and a leading cause of maternal and fetal morbidity and mortality. Although the etiology and pathogenesis of preeclampsia are not fully understood, they are thought to involve maternal, placental, and fetal factors, including abnormalities in uteroplacental vascular development, dysregulated maternal immune responses, and the altered secretion of pro-inflammatory, angiogenic, and anti-angiogenic mediators [104]. Oxidative stress at the maternal–fetal interface, which normally supports placental development by regulating oxidative balance and apoptosis, becomes dysregulated in preeclampsia, contributing to pathological outcomes [105].

In pathological pregnancy conditions, such as preeclampsia, the levels of 8-oxoG are elevated [106–108]. Scaife et al. demonstrated that placental senescence markers—such as p21, phosphorylated histone γH2AX, and 8-oxoG—increase with gestational age. These markers are also significantly elevated in pathological and post-term placentas, suggesting that oxidative stress-induced senescence plays a pivotal role in the progression of preeclampsia [109]. An abnormal maternal immune response to the placenta is a key step in the pathogenesis of preeclampsia, triggering a systemic inflammatory response that impacts endothelial function [110]. Immune cells, such as Th1, Th17, and natural killer (NK) cells, play critical roles in the pathogenesis of preeclampsia. In patients with preeclampsia, increased proportions of pro-inflammatory T cell subsets (Th1 and Th17), along with decreased levels of immunosuppressive populations (Tregs and Th2), have been observed [111, 112]. Furthermore, diminished Treg levels in early pregnancy have been associated with an increased risk of preeclampsia progression [51, 113]. Research conducted by Tadesse et al. indicated that placental tissues from patients with preeclampsia exhibit markedly high levels of OGG1 and APE1. Moreover, in placental tissues affected by oxidative stress, the expression of OGG1 and APE1 in maternal cells (decidua) significantly exceeds that in fetal cells (cytotrophoblasts) [114]. However, the precise role of OGG1 and its downstream products in endothelial oxidative injury and immune activation in eclampsia remains to be elucidated.

### Preterm birth

In addition to being closely associated with various reproductive disorders, oxidative stress is also linked to pregnancy complications, such as preterm birth [108]. In this condition, the inflammatory response at the placental–maternal interface is a crucial factor. The combined effects of inflammatory responses and oxidative stress at the placental–maternal interface contribute to the transformation and senescence of membrane cells. This process, in turn, leads to the release of pro-inflammatory and pro-contractile mediators, ultimately triggering the onset of both term and preterm labor [115]. Menon et al. reported that human placental membranes express OGG1, and that exposure to cigarette smoke induces DNA damage accumulation and elevates basal 8-oxoG levels in these tissues. This highlights the critical role of OGG1 in preserving genomic integrity and preventing preterm birth and premature rupture of fetal membranes [116]. However, current literature on the role of OGG1 in pregnancy complications remains limited and warrants further investigation.

### Cancer risk associated with gynecological diseases

The altered hydrogen-bond donor and acceptor configuration of 8-oxoG, particularly involving protonation of the N7 position, converts it from a hydrogen-bond acceptor to a donor. This shift enables 8-oxoG to form Hoogsteen base pairs with adenine instead of cytosine, leading to its characteristic miscoding and mutagenic potential [117]. In addition to its canonical pairing with cytosine, 8-oxoG can form stable Hoogsteen base pairs with adenine, leading to G:C to T:A transversions during DNA replication [118]. Moreover, OGG1-deficient cells exhibit elevated levels of spontaneous mutagenesis [119]. Furthermore, combined deficiency of OGG1 and MYH glycosylases results in age-related accumulation of 8-oxoG in the DNA of the lungs and small intestine, with cumulative damage in the liver, ultimately increasing cancer susceptibility in *Myh*^*−/−*^*/Ogg1*^*−/−*^ mice [120]. Somatic mutations in the *OGG1* gene are frequently observed in various human cancers, and the gene exhibits a high degree of polymorphism in the general population. Mutant forms of the *OGG1* gene exhibit substantially reduced repair activity relative to that of the wild-type, thereby contributing to different types of tumorigenesis [121]. Moreover, when OGG1 is impaired, the equilibrium between innate and adaptive immune responses is disrupted via heightened oxidative stress and cytokine imbalances, suggesting that this is a crucial target for lung cancer treatment [122]. In gynecologic oncology, polymorphisms in the *OGG1* gene have been linked to the risk and progression of cancers such as ovarian and cervical cancer. Altering OGG1 expression using gene therapy or small-molecule inhibitors can affect ovarian cancer cell growth, apoptosis, and sensitivity to chemotherapy [123, 124]. Additionally, Xu et al. reported that decreased OGG1 expression enhances ultrasound-induced apoptosis in cervical cancer cells [125]. Ba et al. demonstrated that ROS inducers trigger parthanatos in cervical cancer cells through OGG1-catalyzed overexcision of 8-oxoG [80]. Therefore, targeting OGG1 for cancer therapy in obstetric and gynecologic malignancies warrants further in-depth investigation.

## Targeted therapy based on OGG1

TH5487 [40] is a selective small-molecule inhibitor that interferes with the binding of OGG1 to 8-oxoG in DNA, thereby indirectly suppressing OGG1-mediated base excision repair without inhibiting its catalytic activity. This inhibition also decreases DNA occupancy by NF-κB and reduces TNF-α-induced neutrophil recruitment. Furthermore, it downregulates the expression of inflammation-related genes and limits immune cell recruitment, thereby alleviating inflammatory responses in mice. Moreover, treatment with TH5487 significantly inhibits goblet cell hyperplasia, mucus overproduction, and immune cell infiltration in a mouse model of ovalbumin-induced allergic airway inflammation. It also modulates the expression of several key genes associated with asthma pathogenesis [126]. Furthermore, TH5487 treatment leads to an increase in apoptosis and ROS generation in ovarian granulosa cells, alongside a reduction in the inflammatory responses of these cells [96]. Therefore, TH5487-based anti-inflammatory therapy may represent a promising therapeutic approach. Moreover, although oxidative products are elevated in mice lacking OGG1 activity, they do not adversely affect embryonic development, lifespan, or other major physiological outcomes. Instead, inflammation levels are significantly reduced in vivo [127]. These findings suggest that maintaining a balanced level of OGG1 activity may be beneficial for managing inflammatory disorders.

**Fig. 4:**
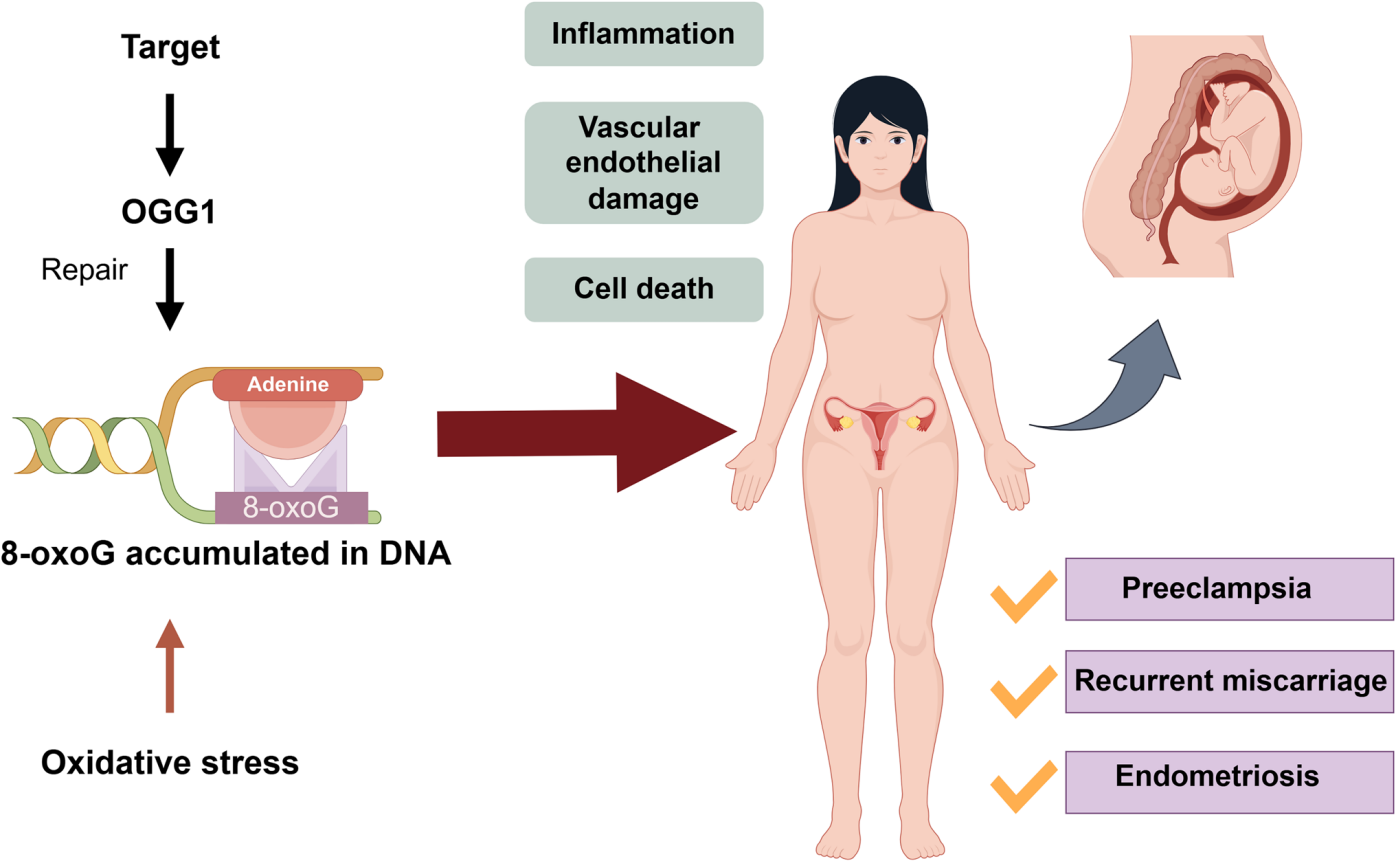
Potential therapeutic role of targeting OGG1 in obstetric and gynecological disorders. Following oxidative stress, 7,8-dihydro-8-oxoguanine (8-oxoG) accumulates in DNA, and OGG1, along with its repair intermediates, can initiate pathological processes, including inflammation, vascular endothelial damage, and cell death. Given its central role in repairing oxidative DNA damage and modulating downstream inflammatory responses, OGG1 has emerged as a promising therapeutic target. Modulating OGG1 activity could offer therapeutic benefits for treating obstetric and gynecological conditions linked to oxidative stress and DNA damage, potentially improving outcomes in disorders such as preeclampsia, recurrent miscarriage, and endometriosis

Antioxidants, such as vitamin C and butylated hydroxyanisole, prevent E2-induced DNA damage in breast carcinogenesis by enhancing the binding of NRF2 to the promoter region of *OGG1* gene [128]. Moreover, OGG1 depletion impairs cancer cell proliferation by inducing S-phase DNA damage, replication stress, and subsequent cell cycle arrest or apoptosis, thereby supporting OGG1 as a potential therapeutic target in cancer. Additionally, OGG1 depletion has been shown to reduce tumor growth in vivo [129]. ROS-inducing agents promote parthanatos in cervical cancer cells by inducing DNA strand breaks via OGG1-mediated excision of 8-oxoG. Furthermore, TH588—a microtubule-interacting agent and selective inhibitor of MTH1—impairs the sanitization of oxidized nucleotides, resulting in mitosis-dependent accumulation of genomic 8-oxodG and disruption of mitotic progression, thereby contributing to tumor growth suppression [80]. This approach may offer a more targeted and safer strategy for cervical cancer chemotherapy. Enzymes such as OGG1 are integral to the pathways that regulate cancer and inflammation, making them promising targets for diagnosis and future therapeutic interventions (Fig. 4).

## Conclusions

OGG1 plays a critical role in obstetric and gynecological diseases by influencing multiple pathological processes, including inflammation, vascular endothelial damage, and cell death. Its functions span DNA repair, tumorigenesis, tumor progression, and pregnancy-related complications. Although we have made every effort to comprehensively review the available literature, we acknowledge that current studies specifically focusing on OGG1 in obstetric and gynecological diseases remain relatively limited. This scarcity of research not only presents a challenge in drawing more definitive conclusions but also highlights a critical knowledge gap in the field. Therefore, our review aims not only to summarize existing evidence but also to draw attention to this underexplored area, which we believe holds significant potential for future discoveries. In-depth investigations into the roles of OGG1 and its underlying molecular mechanisms are urgently needed. Ultimately, advancing our understanding of OGG1 may facilitate the development of novel therapeutic strategies to improve patient outcomes and quality of life in gynecologic and obstetric diseases.

## Data Availability

Data sharing is not applicable to this study, as no datasets were generated or analyzed.
